# Static spatial growth restriction micropatterning of endothelial colony forming cells influences their morphology and gene expression

**DOI:** 10.1371/journal.pone.0218197

**Published:** 2019-06-12

**Authors:** Matthew W. Hagen, Monica T. Hinds

**Affiliations:** Department of Biomedical Engineering, Oregon Health & Science University, Portland, OR, United States of America; INSERM, Université de Bordeaux, FRANCE

## Abstract

**Background:**

Endothelialization of small diameter synthetic vascular grafts is a potential solution to the thrombosis and intimal hyperplasia that plague current devices. Endothelial colony forming cells, which are blood-derived and similar to mature endothelial cells, are a potential cell source. Anisotropic spatial growth restriction micropatterning has been previously shown to affect the morphology and function of mature endothelial cells in a manner similar to unidirectional fluid shear stress. To date, endothelial colony forming cells have not been successfully micropatterned. This study addresses the hypothesis that micropatterning of endothelial colony forming cells will induce morphological elongation, cytoskeletal alignment, and changes in immunogenic and thrombogenic–related gene expression.

**Methods:**

Spatially growth restrictive test surfaces with 25 μm-wide lanes alternating between collagen-I and a blocking polymer were created using microfluidics. Case-matched endothelial colony forming cells and control mature carotid endothelial cells were statically cultured on either micropatterned or non-patterned surfaces. Cell elongation was quantified using shape index. Using confocal microscopy, cytoskeletal alignment was visualized and density and apoptotic rate were determined. Gene expression was measured using quantitative PCR to measure KLF-2, eNOS, VCAM-1, and vWF.

**Results:**

Endothelial colony forming cells were successfully micropatterned for up to 50 hours. Micropatterned cells displayed elongation and actin alignment. Micropatterning increased the packing densities of both cell types, but did not affect apoptotic rate, which was lower in endothelial colony forming cells. KLF-2 gene expression was increased in micropatterned relative to non-patterned endothelial colony forming cells after 50 hours. No significant differences were seen in the other genes tested.

**Conclusions:**

Endothelial colony forming cells can be durably micropatterned using spatial growth restriction. Micropatterning has a significant effect on the gross and subcellular morphologies of both cell types. Further study is required to fully understand the effect of micropatterning on endothelial colony forming cell gene expression.

## Introduction

Durable artificial small-diameter synthetic vascular grafts for the treatment of vascular occlusions represent a critical unmet need in modern medicine. Autologous grafting is the current clinical standard for arterial bypass procedures, however limited availability and donor site complications make a substantial portion of the potential patient population ineligible for autologous vessel harvesting [1]. Given that currently available synthetic biomaterials including expanded polytetrafluoroethylene (ePTFE) show limited patency at diameters less than 6 mm, this leaves many patients without viable alternatives [2,3]. One potential solution to this problem is the use of biomaterials endothelialized *in vitro* prior to implantation. The pre-implant establishment of a functional endothelium is capable of limiting the thrombosis and neointimal hyperplasia which limit patency and lead to graft failure.

One potential autologous source of endothelium is the harvesting of endothelial colony forming cells (ECFCs) from a venous blood draw. ECFCs are outgrowth products of circulating endothelial progenitor cells (EPCs) which can be readily prepared in the laboratory by culturing the mononuclear cell-containing buffy coat of mammalian blood with pro-endothelial growth factors including VEGF [4,5]. The minimally-invasive accessibility of EPCs and the endothelial-like nature of fully differentiated ECFCs make them a promising candidate for tissue engineering applications. Several groups, including our own, have performed proof-of-concept studies showing that mature primary endothelial cell (EC) [6,7] and ECFC [8,9] pre-seeding improve the performance of artificial vascular grafts, suggesting that ECFCs could serve as a cell source for an *in vitro* endothelialization approach.

The primary strength of ECFCs as a tissue engineering tool is their similarity in form and function to mature ECs. As the functional interface between flowing blood and the vascular wall, healthy ECs are responsible for the maintenance of hemostasis as well as the prevention of thrombosis and the management of inflammation. The health of EC subpopulations within an individual is substantially influenced by the mechanical environment in which the cells reside. Early studies of atherosclerosis revealed that plaques are most likely to form at sites where the arterial wall is exposed to non-uniform and oscillatory fluid shear stress, while regions where ECs are exposed to unidirectional non-reversing shear stress are largely spared of atherosclerotic lesions [10]. On a cellular level, both ECs and ECFCs exposed to unidirectional fluid shear stress are seen to elongate in the direction of fluid flow, while those exposed to disturbed flow maintain the rounded, cobblestone shape typical of both cell types in static culture [11,12]. Experiments have revealed a number of beneficial functional consequences of unidirectional fluid shear on ECs *in vitro* and *in vivo* and ECFCs *in vitro*, detectable through changes in gene expression. For example, the downregulation of adhesion molecules such as intercellular adhesion molecule 1 (ICAM-1) and vascular cell adhesion molecule 1 (VCAM-1), which are known to promote early atherogenic inflammation is seen in ECs [13] but not in ECFCs [12], while upregulations of antithrombotic factors including endothelial nitric oxide synthase (eNOS) and tissue factor pathway inhibitor are seen in both ECFCs and ECs [14]. Unidirectional fluid shear stress also drives an upregulation in Krüppel-like factor 2 (KLF-2), a transcription factor believed to be a primary driver of the beneficial fluid shear response seen in the endothelium in ECs [15], however to our knowledge this response has yet to be studied in ECFCs. Notably, computational modeling reveals that the fluid flow within the end-to-side anastomoses most commonly used in vascular bypass procedures is highly non-uniform and oscillatory [16,17]. Thus, grafts pre-endothelialized with either cell type are immediately upon implantation subject to detrimental fluid shear stress limiting their potential clinical benefit.

Importantly, it has been shown that mature EC morphology and function are influenced in static tissue culture through anisotropic substrate micropatterning (reviewed in [18]). Our group and others have extensively used spatial growth restriction micropatterning to confine various cell types to predefined lanes, thus controlling their shape and migration [19–21]. It has been shown that primary mature ECs exhibit both robust cytoskeletal alignment [19,22] and anti-inflammatory changes in gene expression, including downregulations of VCAM-1 and increases in KLF-2 expression [23]. Similarly, many groups have demonstrated their ability to manipulate EC morphology using topographic micropatterning with pitches ranging from 0.5–4 μm [18,24]. Given the substantial influence of fluid shear stress on EC functionality, and the non-uniform oscillatory flow profile of the end-to-side anastomoses in bypass grafting, micropatterning of vascular graft biomaterials has the potential to induce a non-thrombogenic endothelium.

While ECFCs have been shown to have a fluid shear stress response similar to ECs [12] as discussed above, past attempts to micropattern them through spatial growth restriction using alternating lanes of collagen-I and bovine serum albumin (BSA) have proven to be technically infeasible due to ECFCs’ comparatively more robust matrix remodeling abilities [25]. In this study, we demonstrate the use of a food grade block copolymer to prevent ECFC spreading between lanes. We use this technique to test our hypotheses that, similar to mature ECs, micropatterned ECFCs in static culture will display a more elongated morphology and altered gene expression relative to non-patterned ECFCs. Specifically, we expect to see relative upregulations of KLF-2 and eNOS, and downregulations of prothrombotic and adhesion molecules in micropatterned ECFCs relative to non-patterned ECFCs.

## Materials & methods

### Ethics statement

All work necessary for the isolation of the primary cells used in this study was approved by the Oregon National Primate Research Center Institutional Animal Care and Use Committee (IP00000049). Male baboons (*papio Anubis)* were cared for and housed by the Oregon National Primate Research Center staff according to the National Institutes of Health “Guide to the Care and Use of laboratory Animals” by the Committee on Care & use of Laboratory Animal Resources, National Research Council (NIH Publications No. 8023, revised 1978). Detailed descriptions of the housing, care, and humane euthanasia of these primates has been published previously [8].

### Cell isolation and culture conditions

#### ECFC isolation

ECFCs were isolated from the blood of male juvenile *Papio anubis* baboons using a previously established protocol [26]. Briefly, 50 mL of venous blood was drawn into a 7% ACD solution. After no more than 45 minutes, blood was mixed 1:1 with Hank’s Buffered Saline Solution (HBSS, Corning) and carefully layered on top of Histopaque-1077 (Sigma). The tubes were then centrifuged (800 G, 30 minutes, without braking) to isolate the buffy coat containing mononuclear cells. This mononuclear cell suspension was isolated, washed and plated on tissue culture plastic pre-coated with 50μg/mL fibronectin (Sigma). Cultures were fed daily for the first seven days of culture, and every other day for up to four additional weeks with EGM-2 (Lonza) + 18% fetal bovine serum (FBS, Hyclone). When colonies of cobblestone-shaped cells appeared, they were isolated by manually scraping the non-colony-occupied portions of wells and passaging with TrypLE (Gibco). These cells were expanded in T-25 flasks as described below, and upon reaching confluence, were passaged, sorted using anti-CD31 magnetic dynabeads (Invitrogen), and expanded in T-150 flasks, as described below.

#### EC isolation

Primary carotid ECs for use as a reference group were isolated as described previously [27] from animals from which ECFCs were previously collected. This study includes case-matched ECs and ECFCs from two animals. Briefly, whole carotid arteries were rapidly excised from male juvenile *Papio anubis* baboons at the time of necropsy, and flushed with and stored in ice-cold Dulbecco’s phosphate buffered saline (PBS, 0.1M, Gibco) containing 2–4% penicillin-streptomycin (PS, Gibco) for 1–4 hours. To release ECs from the vessel wall, arteries were clamped shut at bottom, and filled with a 37°C solution of 600U/mL collagenase type II (Worthington Biochemical) dissolved in endothelial basal medium (EBM, Lonza) and incubated for five minutes. EC-containing collagenase solution was dripped directly into tissue culture wells which were precoated with 50 μg/mL collagen-I from rat tail (Corning) and filled with EGM-2 supplemented with 18% FBS. Cultures were fed every other day with EGM-2 + 18% FBS for up to four weeks until EC colonies occupying at least half of the well were seen. These colonies were passaged using TrypLE, sorted using anti-CD31 magnetic dynabeads (Invitrogen), and expanded in T-150 flasks. Between isolation and experiments, cells were maintained in cryogenic storage.

#### Culture conditions

After isolation, cell cultures were maintained in endothelial growth medium (EGM-2, Lonza) supplemented with 8% fetal bovine serum for a total of 10%. Unless otherwise specified, all cell expansions occurred in tissue culture flasks (Corning) pre-coated with collagen-I from rat tail (BD Biosciences). All cell culture experiments used EGM-2 supplemented with 8% FBS. During expansions and experiments, cells were kept in a sterile, humidified incubator at 37°C and 5% CO_2_.

### Preparation of experimental substrates

Spatially restricted micropatterned culture substrates were prepared using a modified version of a previously established protocol [20]. All steps are performed at room temperature inside a sterile biosafety cabinet. 50 μg/mL collagen-I from rat tail dissolved in 2N acetic acid was pulled through a 2x1 cm polydimethylsiloxane (PDMS) stamp with 25 μm-wide lanes separated by 25 μm. The collagen was allowed to adsorb to non-tissue culture treated polystyrene for at least one hour before excess collagen was aspirated, micro-channels rinsed with PBS, and PDMS stamps removed. Substrates were further rinsed with PBS and then blocked with 0.2% Pluronic F108NF Prill Poloxamer 338 (BASF) in water for at least one hour. The substrates were rinsed and stored in PBS at room temperature for no more than one month before use. Control, non-patterned substrates were prepared using a 60 μL drop of collagen-I in place of PDMS stamps.

### Micropatterning experiments

Animal-matched ECs and ECFCs were seeded onto micropatterned substrates at 4.55x10^3^ cells / cm^2^ and allowed to attach for one hour. Non-attached cells were then washed away and cultures were fed. Cultures were maintained without additional feeding for either 24 or 50 hours before processing. Where noted, cells were challenged using 100 U/mL tumor necrosis factor α (TNFα, R&D Systems) via a 1/3 feed for two hours prior to experimental end points. Non-TNFα treated samples were given a 1/3 feed of unmodified media at the same time point.

### Quantifying morphology

Morphology was characterized as previously described [19]. Briefly, cultures were imaged live after 22 or 48 hours (immediately prior to TNF-α stimulation) using a phase-contrast enabled 20X objective on a Nikon Eclipse TE 2000-U microscope. Two images per culture were captured and given to a blinded volunteer who manually traced 5–10 cells per image using a freehand tracing tool in Image J software (NIH). Shape index (SI) was calculated as a ratio of cell surface area to perimeter: $SI=\frac{4\pi (SA)}{P^{2}}$. One biological replicate in the 50 hour micropatterned EC group was excluded due to poor patterning likely resulting from a defective PDMS stamp used in substrate preparation.

### Immunofluorescence and super-resolution microscopy

Cells were fixed in 3.7% PFA for fifteen minutes and stored in PBS with Ca^++^ and Mg^++^ (Gibco) at 4°C until further processing. Cells were permeabilized with 0.1% Triton and blocked with FX signal enhancer (Invitrogen). Stains, antibodies, and further blocks were incubated at room temperature for the following times in the following order: Phalloidin (Invitrogen, 1:200, 1 hr), 10% normal goat serum (ThermoFisher, 30 min), Mouse IgG anti VE-cadherin (Santa Cruz Biotechnology, mouse IgG1 monoclonal, clone F-8, Lot 1605, 1:100, 1 hr), Alexa-488 anti Mouse IgG H+L (Invitrogen, goat polyclonal, lot 41786A, 1:500, 30 min), DAPI (Invitrogen, 1:40000, 5 min). Stained samples were mounted with ProLong Gold Antifade (Invitrogen). In order to enable imaging on an inverted confocal system, coverslipped bottoms were cut out of culture dishes and adhered to microscope slides using nail polish (Covergirl).

All imaging was performed using a Zeiss LSM 880 inverted confocal microscope system. Low-magnification IF images were collected using a 10X PlanApo objective NA = 0.45. Z-stacks are presented in figures as maximum intensity Z-projections. Super-resolution microscopy (SRM) imaging was accomplished using a 20X PlanApo objective NA = 0.8 and Fast Airyscan detector array. SRM images were collected as single-slices in order to observe cytoskeletal elements at a resolution of 7nm^2^ / pixel. Automatic airy processing was performed using Zen Black (Zeiss), and post-processing of all images was performed using FIJI (SciJava) [28].

### Quantifying cell density and rates of apoptosis

Cell density and apoptotic rate were quantified using an unbiased counting method and systematic random sampling. Four samples per combination of cell type (EC, ECFC), micropattern, and culture time (24 hour, 50 hour) were fixed and stained as described above. Three high-resolution low-magnification 10X confocal 850 μm x 850 μm images per sample were collected as described above. These images were imported into FIJI and a random grid with 10,000 μm^2^ gridcells was generated over the image using FIJI’s grid tool. Starting in the top left of the image, counting frames were defined as the top left cell in each 2x2 group, such that 16 counting frames per image were selected. Within each counting frame, cell nuclei were counted as either healthy or pyknotic (dense and/or fractured in appearance). All frames were counted by a single blinded individual. In order to ensure the accuracy of the density estimate, only nuclei which were either entirely contained in the counting frame or were partially touching the bottom or left edges of the counting frame were counted. Total cell density is defined as healthy + pyknotic nuclei, while percent apoptotic cells is defined as the ratio of pyknotic to total counted nuclei. To enable an accurate comparison between micropatterned and non-patterned cell densities, counted micropattern densities were doubled to account for the fact that only half of the surface area was available for cell attachment. For the purposes of statistical analysis, each culture trial was considered one biological replicate.

### Quantifying gene expression

Gene expression was quantified using quantitative real-time polymerase chain reaction (qPCR) as previously described [26]. Cells were passaged from micropatterned or non-patterned control substrates using TrypLE, resuspended in Buffer RLT (Qiagen) with 1% β-mercaptoethanol. RNA was extracted from cells using an RNEasy Mini-prep kit (Qiagen) according to manufacturer instructions. RNA yield was determined using Nanodrop (Thermo scientific) and normalized within culture trial to between 25–35 ng RNA before proceeding with reverse transcription. RNA was treated with DNAse I (Fermentas) and RNA reverse transcribed to cDNA using SuperScript III Reverse Transcriptase (Life Technologies) with random primers according to manufacturer protocol. qPCR was performed using Platinum SYBR Green qPCR SuperMix-UDG, ROX reference dye and either the 7500 Fast Real Time PCR system or the QuantStudio 3 (Applied Biosystems, both). Gene expression was determined using previously validated primers [23,26] as summarized in Table 1. Following runs, a well-by-well quality check was performed based on melt curves and variation between technical replicates; samples were excluded when melt points differed from predicted melt points, multiple melt points appeared, no amplification was observed, or significant variation between technical replicates was observed. While sample numbers were always balanced during study design and execution, sample exclusion at this stage frequently resulted in unbalanced sample sizes at the time of data analysis. Changes in gene expression were calculated using the ΔΔCT method, with Glyceraldehyde 3-phosphate dehydrogenase (GAPDH) serving as a housekeeping gene for the calculation of ΔCT, and a control group of non-patterned, non-TNF-stimulated, 24 hour-cultured ECFCs for the calculation of ΔΔCT. -ΔΔCT values are reported in figures and in text, and ΔCT values were used for statistical comparisons of groups. For the purposes of statistical analysis, each culture dish was considered a biological replicate.

**Table 1:** Primers used for quantification of EC and ECFC gene expression.

Gene	Forward	Reverse
**GAPDH**	CCTCAACGACCACTTTGTCA	TTACTCCTTGGAGGCCATGT
**VCAM-1**	GGGAAGATGGTCGTGATCCTT	TGAGACGGAGTCACCAATCTG
**vWF**	CCTATTGGAATTGGAGATCGCTA	CTTCGATTCGCTGGAGCTTC
**KLF-2**	CACCGGGTCTACACTAGAGG	AAATGCCGCAGACAGTACAA
**eNOS**	ATCTCCGCCTCGCTCATG	AGCCATACAGGATTGTCGCCT

Forward and reverse primer sequences for VCAM-1, vWF, KLF-2, and eNOS. All primers used in this study were previously validated [23,26].

### Statistics

Results are reported as mean ± standard deviation. All statistics were calculated using R (R foundation for statistical computing, version 3.5.1) [29], and R packages Infer (version 0.3.0) [30], and multcomp [31]. Comparisons of cell density and apoptotic rate were performed using multi-way ANOVAs with coefficients for micropatterning, culture time, cell type, and interactions as appropriate. Where interactions were involved, specific between-groups differences were calculated using the general linear hypothesis test based on manually constructed linear combinations, and multiple-comparison p-values corrected using the Holm method. Because each factor contained only two levels, post hoc testing was not necessary when no statistically significant interactions were involved. The appropriateness of parametric methods was confirmed by ensuring balanced sample sizes, and examining model quantile-quantile plots and fitted versus residual value plots.

Because of unbalanced sample sizes and the non-normal distributions of shape index and qPCR data, these comparative statistical tests were performed using two-sample, one-tailed, 10,000 replicate permutation tests [32]. These nonparametric one-tailed tests were set up to test the specific hypotheses addressed here, specifically, that micropatterning reduces shape index, VCAM-1, and vWF gene expression, and increases KLF-2 and eNOS gene expression. For all results displayed, each culture dish is considered a biological replicate. Results were considered statistically significant when p < 0.05.

## Results

### Polymer-based method effectively spatially restricts ECFC growth into micropatterned lanes

**Fig 1:**
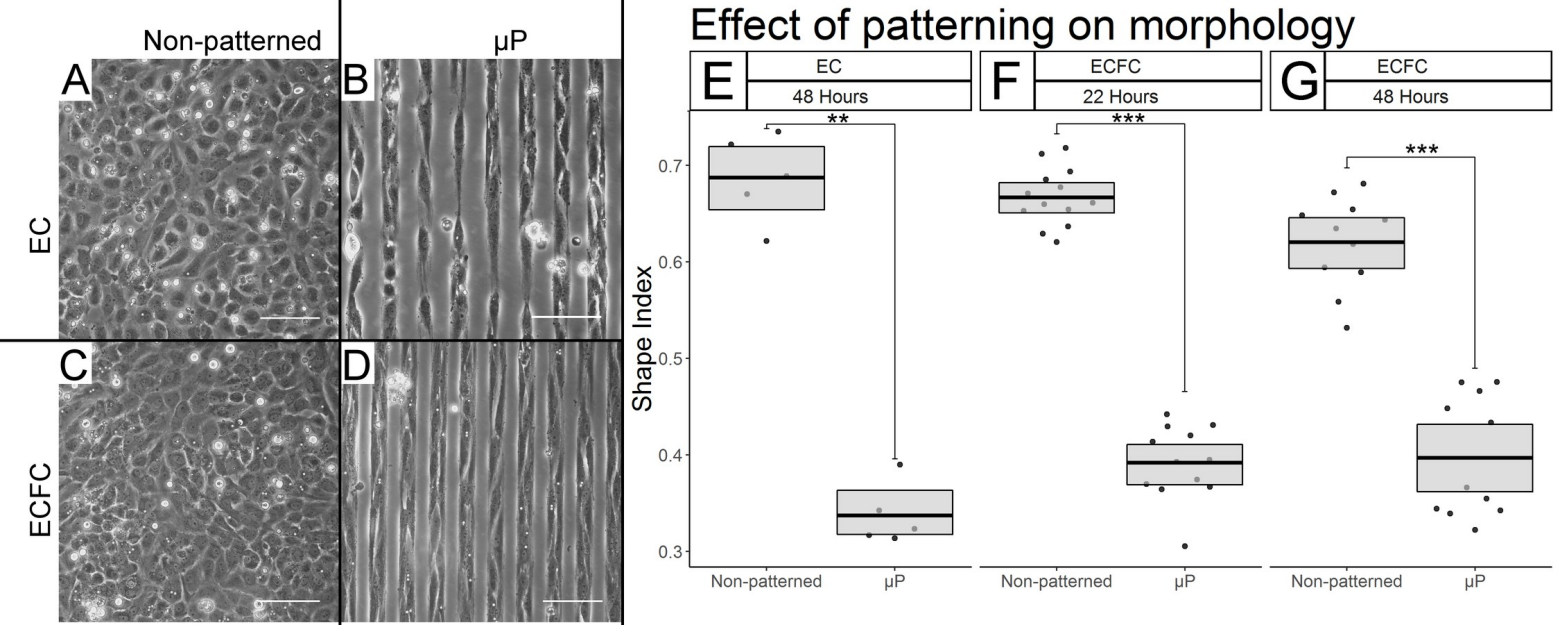
Spatial growth restriction micropatterning effectively growth restricts ECFCs and affects their gross morphology. Representative phase contrast light microscopy after 48 hours of culture at 20X (A-D) reveals effective growth restriction in ECs (B vs. A), and ECFCs (D vs. C) Scale bars = 100 μm. (E-G) Shape index quantification of EC and ECFC elongation. Box limits represent bootstrapped 95% confidence intervals, and crossbars represent means. Each point represents the mean SI of the 5–10 cells traced from each biological replicate. We find statistically significant micropatterning effects in ECs after 50 hours (E, p = 0.0052), and in ECFCs after 24 (F, p < 0.0001) and 50 hours (G, p < 0.0001).

First the ability of the modified micropatterning procedure to effectively spatially restrict ECFC growth on tissue culture plastic was assessed (Fig 1). Live phase-contrast light microscopy revealed well-defined lanes of ECFCs on patterned substrates (Fig 1D) with minimal crossover occurring at both 22 and 48 hours, suggesting that the modified procedure did effectively spatially growth-restrict ECFCs, to an extent similar to that seen in micropatterned ECs (Fig 1B)

### Spatial growth restriction micropatterning has a substantial effect on ECFC elongation

After the efficacy of growth restriction was confirmed, the effect of micropatterning on the shape of individual ECFCs was assessed using shape index (SI), a measure of roundness, calculated as a ratio of surface area to perimeter such that SI = 0 represents a straight line and SI = 1 a perfect circle. After 22 hours, a dramatic reduction in SI was observed in micropatterned (μp) relative to non-patterned ECFCs from 0.667 ± 0.148 to 0.393 ± 0.132 (Fig 1F, p <0.0001, N = 13 (non-patterned), 12 (μp)). This difference in shape index was maintained after 50 hours of culture time (Fig 1G, p < 0.0001, non-patterned = 0.626 ± 0.155, μp = 0.362 ± 0.132, N = 11 each). The SI of micropatterned and non-patterned ECs was also calculated as a reference point after 50 hours for reference and showed a nearly identical degree of elongation (Fig 1E, p = 0.0052, non-patterned = 0.683 ± 0.154, μp = 0.337 ± 0.111, N = 5 each).

### Micropatterning drives alignment of the ECFC actin cytoskeleton

**Fig 2:**
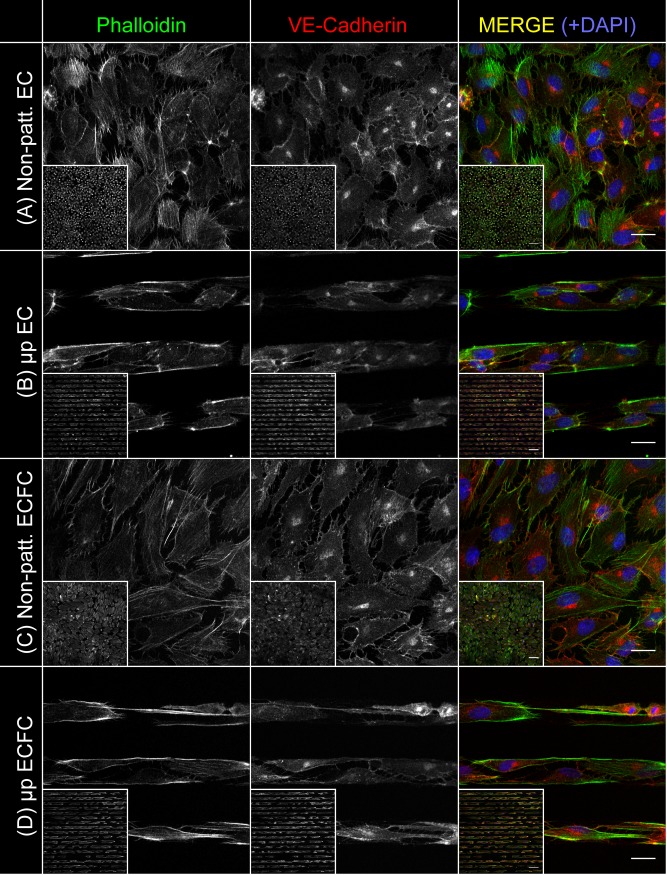
Micropatterned cells show subcellular alignment and peripheral aggregation of actin. ECFCs and ECs were stained and imaged using laser scanning confocal microscopy with a super-resolution airyscan detector in order to assess actin distribution and alignment after 24 hours of culture. Representative images of non-patterned ECFCs (A), micropatterned ECFCs (B), non-patterned ECs (C), and micropatterned ECs (D) after 24 hours in culture are shown. Each image series shows phalloidin (actin), VE-Cadherin, and a pseudocolor merge with phalloidin in green, VE-Cadherin in red, and a DAPI nuclear stain in blue. Main panel scale bars are 20 μm. Inset panels are 10X maximum Z-projections, with scale bars of 100 μm.

The effect of micropatterning on the subcellular architecture of ECFCs was characterized after 24 hours of culture time. Super-resolution imaging of the actin cytoskeleton of non-patterned ECFCs showed random organization of actin fibers (Fig 2A), whereas micropatterned ECFC actin fibers were aligned (Fig 2B). A similar degree of relative alignment was seen in micropatterned ECs relative to non-patterned ECs (Fig 2C and 2D). Non-patterned ECs and ECFCs had a uniform distribution of actin fibers throughout the cytoplasm, whereas both cell types displayed a concentration of actin at their peripheries when micropatterned. Interestingly, although all cultures were seeded to confluence, some interruptions in the VE-Cadherin cell-cell contacts were visible in all conditions examined.

### Micropatterning increases EC and ECFC density, but has no effect on apoptotic rate

**Fig 3:**
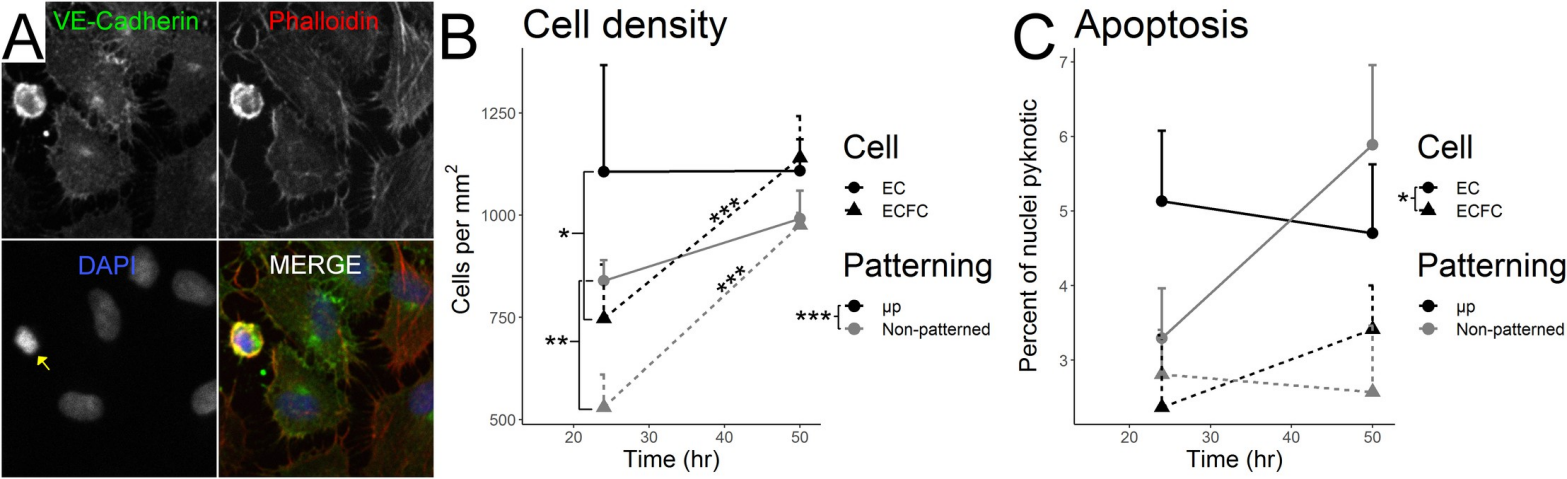
Micropatterning significantly increases cell density. **EC apoptotic rate is higher than ECFC, regardless of micropatterning.** Stained EC and ECFC nuclei were counted in order to quantify cell density and apoptotic rate. (A) Representative micrograph of a pyknotic nucleus (yellow arrow, DAPI channel) surrounded by healthy cells in a non-patterned EC sample after 50 hours of culture. (B) Micropatterning significantly increases cell density across other culture conditions (p = 0.0002). Post-hoc testing showed significantly greater EC than ECFC density at 24 hours in both non-patterned (p = 0.0024) and micropatterned cells (p = 0.011), however no significant cell-type differences existed after 50 hours (p = 1, both). As noted by stars above trend lines, ECFC density significantly increased between 24 and 50 hours of culture time in both non-patterned (p = 0.0009) and micropatterned (p = 0.0002) cultures, while EC density did not (p = 1 non-patterned, p = 0.689 μp). (C) ECs showed a significantly greater rate of apoptosis than ECFCs across culture conditions (p = 0.0151), however micropatterning (p = 0.732) and culture time (p = 0.336) had no significant effects on apoptotic rate.

EC and ECFC density and apoptotic rate were quantified by counting nuclei in stained cultures (Fig 3A). Micropatterning significantly increased the densities of both cell types (Fig 3B, ANOVA F_1,24_ = 20.21, p = 0.0002). Cell type also significantly affected density (ANOVA F_1,24_ = 14.68, p = 0.0008), as did culture time (ANOVA F_1,24_ = 34.13, p = 5.03x10^-6^), however these factors significantly interacted (ANOVA F_1,24_ = 16.22, p = 0.0005). After 24 hours of culture time, non-patterned ECs were more densely packed than non-patterned ECFCs (EC 839 ± 51 cells / mm^2^, ECFC 530 ± 80 cells / mm^2^; p = 0.0024), and micropatterned ECs were likewise more densely packed than micropatterned ECFCs (EC 553 ± 130 cells / mm^2^, ECFC 373 ± 66 cells / mm^2^; p = 0.011), while after 50 hours of culture time there was no significant difference between non-patterned ECs and ECFCs (EC 992 ± 68 cells / mm^2^, ECFC 979 ± 29 cells / mm^2^, p = 1), or micropatterned ECs and ECFCs (EC 554 ± 39, ECFC 570 ± 51 cells / mm^2^, p = 1). The density of ECs did not change significantly between 24 and 50 hours of culture time regardless of micropatterning (p = 1 non-patterned, p = 0.689 μp, see means above), while the density of ECFCs increased significantly under both conditions (p = 0.0009 non-patterned, p = 0.0002 μp, see means above).

A greater portion of ECs were apoptotic compared to ECFCs across culture conditions (Fig 3C, ANOVA F_1,28_ = 6.71, p = 0.0151), while micropatterning and culture time had no significant effects (micropatterning F_1,28_ = 0.12, p = 0.732; culture time F_1,28_ = 0.958, p = 0.336) with no interactions (at 24 hours: non-patterned ECs 3.29 ± 1.7% apoptotic, μp ECs 5.13 ± 2.6%, non-patterned ECFCs 2.81 ± 1.7%, μp ECFCs 2.37 ± 1.1%. At 50 hours: non-patterned ECs 5.89 ± 2.8% apoptotic, μp ECs 4.7 ± 3.3%, non-patterned ECFCs 2.57 ± 1.3%, μp ECFCs 3.41 ± 2.2%.).

### Micropatterning has a modest effect on ECFC transcriptional regulation over time

**Fig 4:**
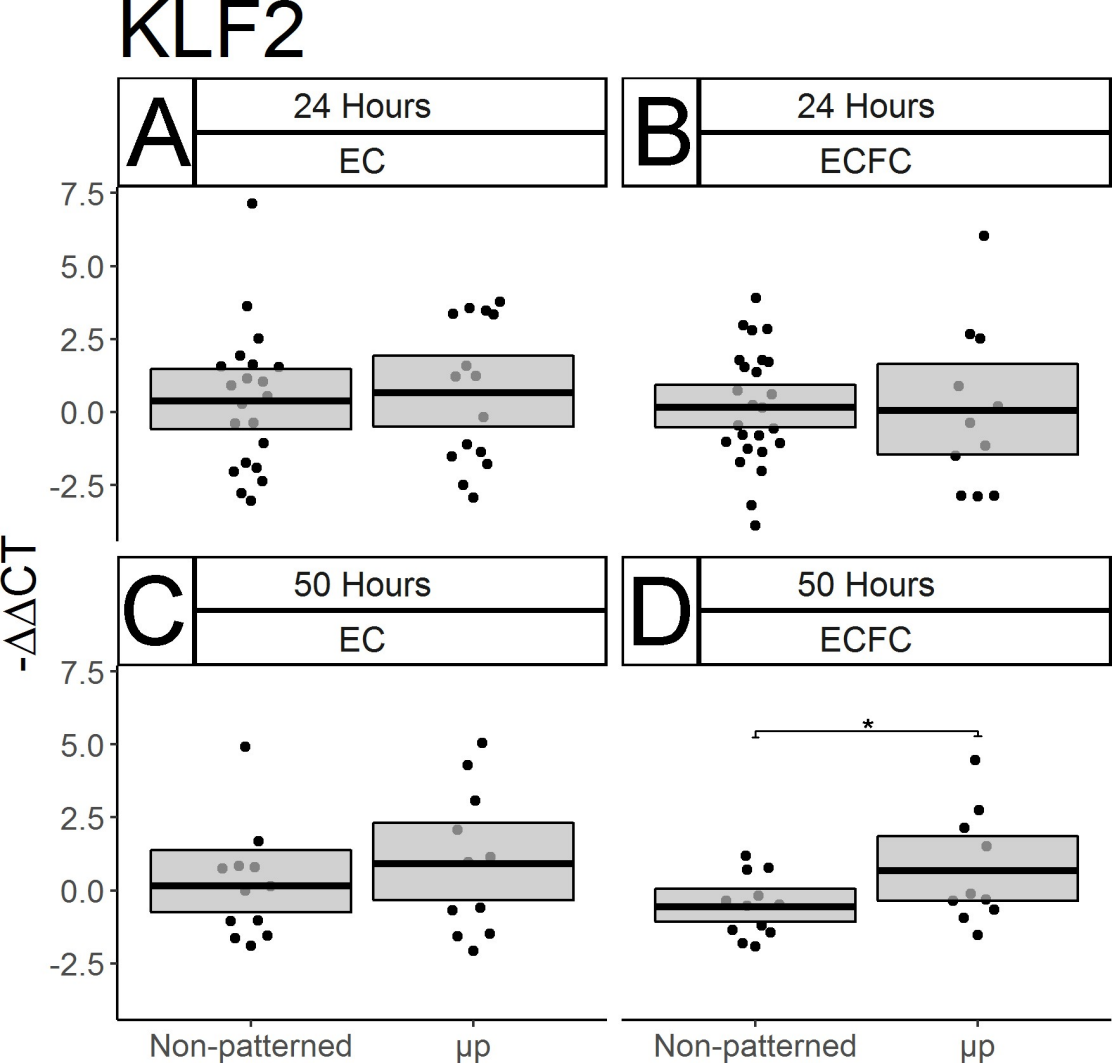
Micropatterning has a time-dependent effect on ECFC transcriptional regulation. The expression of the transcriptional regulator KLF-2 was measured in ECs and ECFCs after 24 and 50 hours using qPCR. Results are displayed as–ΔΔCT. Box limits represent bootstrapped 95% confidence intervals. Each point represents one biological replicate. No effect of micropatterning on EC KLF-2 expression was seen after 24 or 50 hours (A, C). Nor was any change seen in ECFCs after only 24 hours (B, p = 0.550), however a significant increase in KLF-2 expression was seen in ECFCs after 50 hours (D, p = 0.0328).

After demonstrating the effectiveness of spatial growth restriction on the gross and subcellular morphology of ECFCs, qPCR was used to determine its effect on KLF-2, which is recognized as a master regulator of the EC response to unidirectional FSS *in vivo*. After 24 hours, ECFCs showed no discernible micropatterning effect on KLF-2 expression (Fig 4B, p = 0.550, N = 25 non-patterned, 11 μp), however a statistically significant effect did appear after 50 hours of culture time (Fig 4D, p = 0.0328, N = 12 non-patterned, 10 μp), suggesting that micropatterning did encourage an increase in KLF-2 expression at the later time point. Interestingly, no significant effect of micropatterning was seen on EC expression of KLF-2 at either time point (Fig 4A,C, for 24 h: p = 0.327; N = 21 non-patterned, 15 μp; for 50h: p = 0.211; N = 12 non-patterned, 11 μp).

### eNOS gene expression is not significantly affected by micropatterning

**Fig 5:**
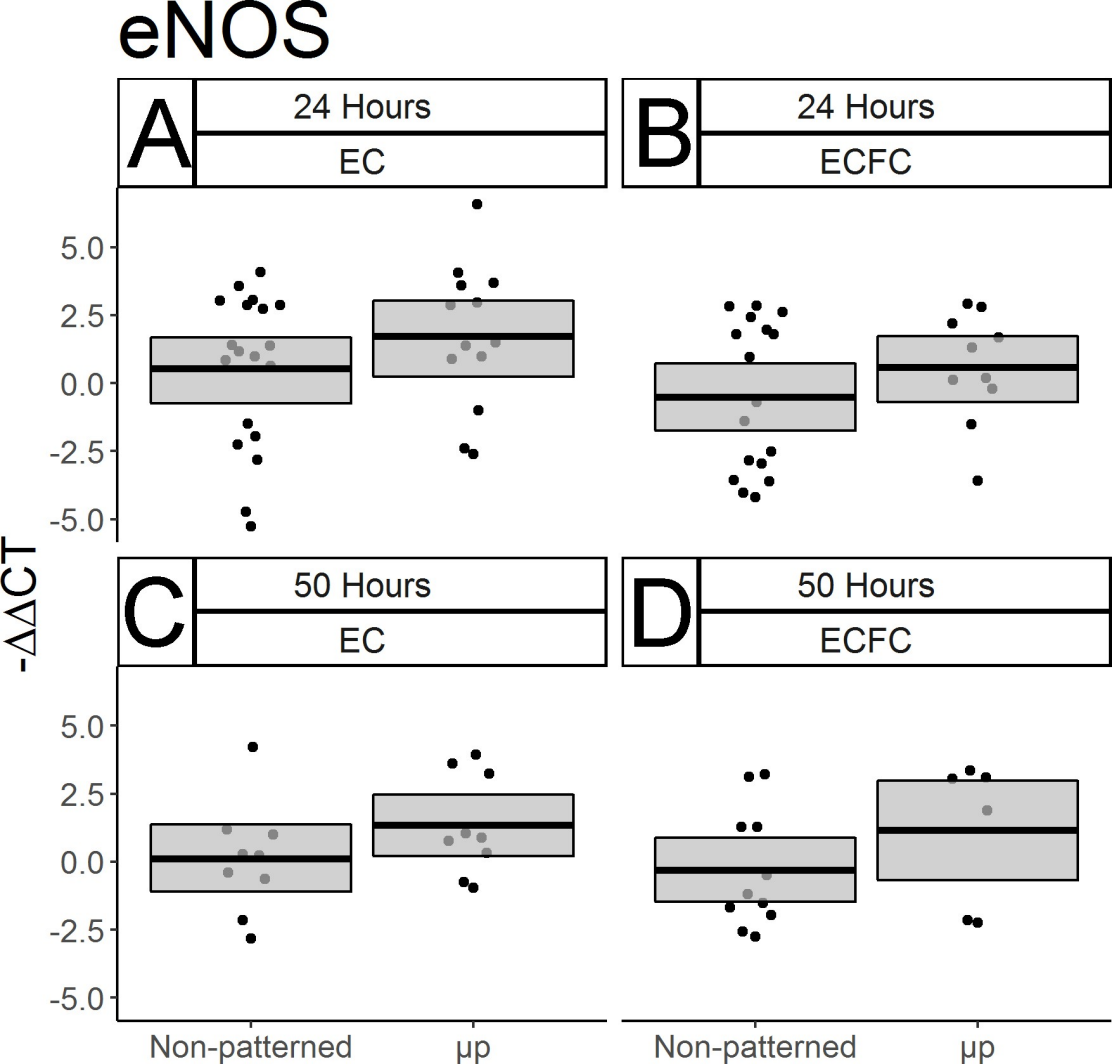
ECFC eNOS gene expression after micropatterning. qPCR was used to measure EC and ECFC gene expression of eNOS with and without micropatterning after 24 and 50 hours of culture. Results are displayed as–ΔΔCT. Box limits represent bootstrapped 95% confidence interval. Each point represents one biological replicate. In both cell types, and at both time points measured, no statistically significant differences in eNOS expression were seen in response to micropatterning. (A) ECs after 24 hours. (B) ECFCs after 24 hours. (C) ECs after 50 hours, (D) ECFCs after 50 hours.

Given the importance of nitric oxide as a vasodilator and antiplatelet agent, the effect of micropatterning on the expression of the endothelial nitric oxide synthase (eNOS) gene was also measured. No statistically significant effect of micropatterning was seen at either time point in ECFCs (Fig 5B and 5D. For 24h: p = 0.1423; N = 17 non-patterned, 10 μp. For 50h: p = 0.1169; N = 11 non-patterned, 6 μp). or ECs (Fig 5A and 5C: For 24h: p = 0.1199; N = 20 non-patterned, 13 μp. For 50 h: p = 0.0986; N = 9 each).

### Micropatterning did not significantly impact pro-inflammatory and pro-thrombotic gene expression by ECFCs

**Fig 6:**
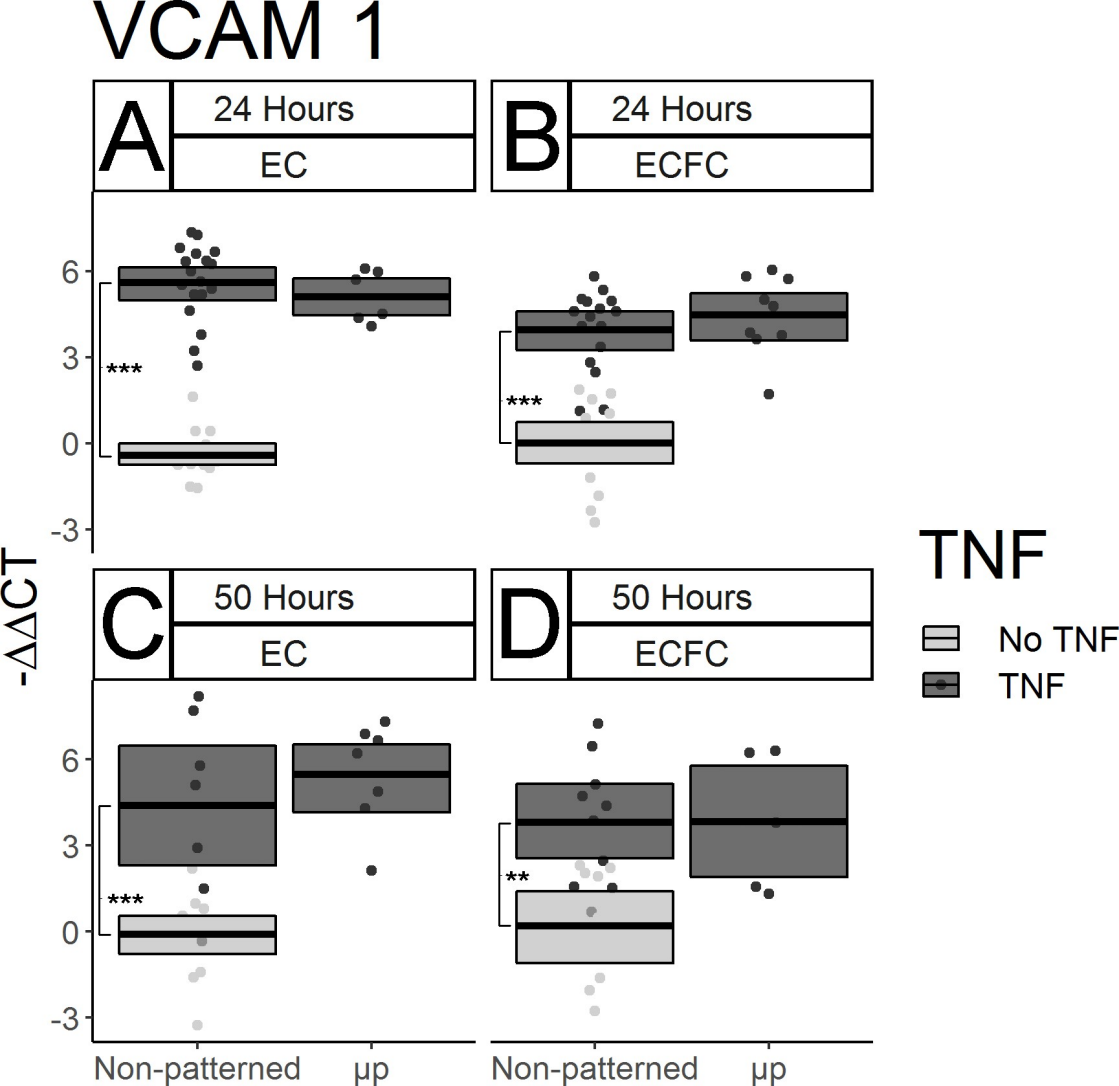
Micropatterning has no discernible effect on inflammatory molecule gene expression in ECs or ECFCs. VCAM-1 gene expression was measured by qPCR in ECs and ECFCs after 24 and 50 hours of culture. Data are displayed as–ΔΔCT. TNF-α was added for the final two hours of culture. Box limits represent bootstrapped 95% confidence intervals and crossbars represent means. Each point represents one biological replicate. Light gray boxes and points represent gene expression for non-patterned, non-TNF treated cells in each cell type and time point. TNF-α efficacy at stimulating increased VCAM-1 gene expression was confirmed in each cell type at both time points. (A) ECs after 24 hours (p < 0.0001). (B) ECFCs after 24 hours (p < 0.0001). (C) ECs after 50 hours (p < 0.0001). (D) ECFCs after 50 hours (p = 0.0003). No micropatterning-dependent difference in TNF-α stimulated VCAM-1 expression was seen in either cell type at either time point.

**Fig 7:**
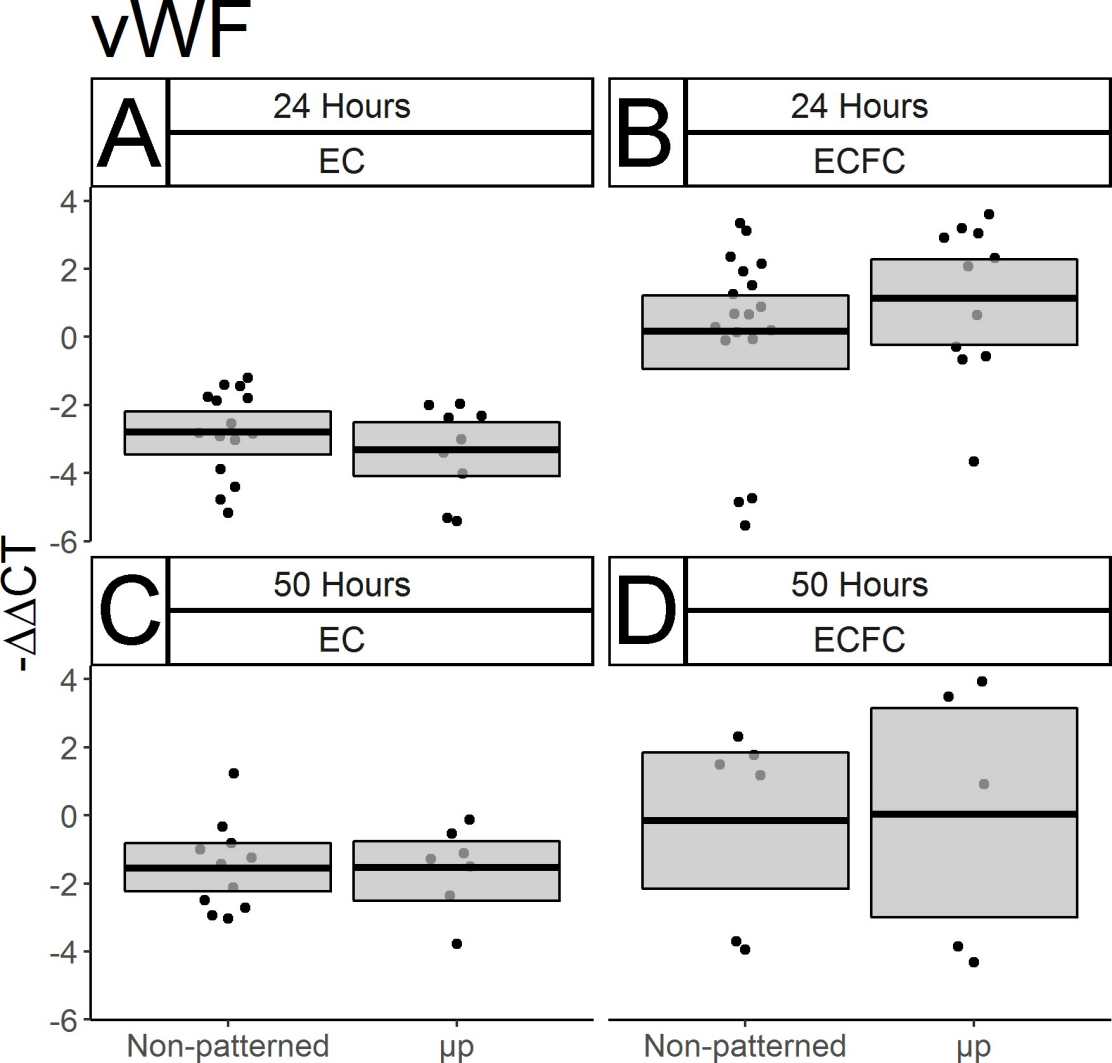
Micropatterning has no discernible effect on the expression of the prothrombotic von Willebrand factor in ECs or ECFCs. vWF gene expression was measured by qPCR in ECs and ECFCs after 24 and 50 hours of culture. Results are displayed as–ΔΔCT. Box limits represent bootstrapped 95% confidence intervals, and crossbars represent means. Each point represents one biological replicate. No statistically significant micropatterning-driven differences were seen in any of the conditions tested: (A) ECs after 24 hours, (B) ECFCs after 24 hours, (C) ECs after 50 hours, (D) ECFCs after 50 hours. Notably, an increase in vWF expression was seen in ECFCs over ECs after 24 hours (A vs. B).

The effect of spatial growth restriction micropatterning on pro-inflammatory and pro-thrombotic gene expression by mature ECs and ECFCs was also characterized. In order to amplify the baseline gene expression of the pro-inflammatory gene VCAM-1, cells were treated with 100 U/mL TNF-α prior to collection and RNA isolation. Substantial increases of VCAM-1 expression in TNFα-treated non-patterned ECs relative to untreated non-patterned ECs after 22 hours of culture and two hours of cytokine treatment (Fig 6A, p < 0.0001, N = 16 non-treated, 18 TNF-α treated), and after 48 hours of culture and two hours of cytokine treatment (Fig 6B, p = 0.0004, N = 14 non-treated, 7 TNF-α treated) were observed. This increase was likewise seen in ECFCs after 22 hours of culture and two hours of TNF-α treatment (Fig 6B p < 0.0001, N = 14 non-treated, 16 treated) and after 48 hours of culture and two hours of TNF-α treatment (Fig 6D, p = 0.0003, N = 14 non-treated, 7 treated). No effect of micropatterning was seen on VCAM-1 expression in either cell type or time point (Fig 6A–6D, p > 0.2, N between 5–18 throughout). Expression of the gene encoding von Willebrand Factor (vWF), a molecule secreted by ECs *in vivo* which, among other things, recruits platelets to a nascent clot was also measured. No noticeable micropatterning-driven differences were seen after the first 24 hours of culture (Fig 7A, p > 0.1, N between 9–18, both), or after 50 total hours of culture (Fig 7B, p > 0.1, N between 5–11, both). Interestingly, there was evidence of a cell type effect after 24 hours, with non-patterned ECFCs expressing more of the gene than ECs (Fig 7A vs.7B), however the extent of this difference decreased after 50 hours of culture (Fig 7C vs. 7D). Because this difference was not among the hypotheses we designed the study to test in the original experimental design, this difference was not tested statistically.

## Discussion

To our knowledge, this study represents the first successful demonstration that ECFC morphology and gene expression can be manipulated in static culture. While the ECFC literature contains conflicting reports on the degree of similarity between ECFCs and mature ECs [5], in our non-human primate model, ECFCs are remarkably similar to ECs in morphology and function [8,14,33,34]. Our earlier studies revealed similar expression levels of the genes and cell surface proteins that regulate monocyte adhesion, tight junction formation, and platelet activation between ECs and ECFCs both basally and following TNF-α stimulation [27]. We furthermore previously showed similar performances of EC and ECFC-seeded vascular grafts in our *ex vivo* shunt model of platelet and fibrin accumulation [27]. However, ECs and ECFCs do have a few notable differences: ECFCs have a much lower basal level of eNOS gene expression [27], and are capable of higher rates of proliferation and extracellular matrix remodeling in culture than ECs [25,34]. We have hypothesized that this latter difference is the primary reason that a prior attempt to micropattern ECFCs was not successful: the use of BSA to block ECFC expansion between patterned lanes was not sufficient to overcome the enhanced proliferation and remodeling ability of these cells [25]. Through the use of an increased concentration of Pluronic, a previously validated [20] food-grade hydrophobic polymer, we have created the conditions for durably elongated ECFCs under static culture conditions for longer periods of time.

In further characterizing the effect of micropatterning on the morphology of our ECFCs, we have seen highly significant and consistent cell elongation, as represented by the decreased shape indices shown in Fig 1. These results were both internally consistent with the case-matched ECs we observed in this study, and with the SI values of mature statically micropatterned ECs and *in vivo* flow-elongated ECs reported on previously [19]. Confocal microscopy was used to demonstrate that the degree of alignment in the actin cytoskeletons of our ECs and ECFCs was similar. Imaging of the actin cytoskeleton further revealed the high concentration of the actin cytoskeleton into dense peripheral bands, as seen previously in micropatterned ECs [19].

We also quantified the density and apoptotic rates of ECs and ECFCs with and without micropatterning at multiple time points. As shown in Fig 3, we found that both ECs and ECFCs were more densely packed when seeded onto micropatterned rather than non-patterned surfaces. This arguably contradicts an early study of mature EC growth restriction which found that proliferation decreases with the width of the ECM substrate on which cells are allowed to grow [35]. However, these earlier studies measured tritiated thymidine uptake, an indicator of active proliferation, whereas we measured static density. Interestingly, we found cell-type specific effects of both density and apoptotic rate. Regardless of micropatterning, ECFC density increased between 24 and 50 hours, while no significant changes were seen in EC density over the same period. Additionally, ECs showed a consistently greater rate of apoptosis over ECFCs regardless of micropatterning or culture time, suggesting that ECFCs have both a greater proliferative potential and resilience against culture conditions than ECs.

In addition to demonstrating the efficacy of our spatial growth restriction technique on the modulation of ECFC morphology, density, and survival, we used qPCR to approximate the function of micropatterned ECFCs by measuring the expression of key genes. We showed that after two days of micropatterned culture, the expression of KLF-2, a master regulator of EC responses to fluid shear stress *in vivo* [36], was significantly elevated in ECFCs relative to non-patterned ECFCs, suggesting that ECFC gene expression, as well as morphology, can be manipulated statically through spatial growth restriction micropatterning. However we did not see any significant effect of micropatterning on the expression of eNOS, an antithrombotic factor which is basally under-expressed in ECFCs [34].

While the results of our gene expression study do show some support of our hypothesis that static micropatterning will drive resistance to immunogenic and thrombogenic stimuli in the form of increased KLF-2 expression, we observed notable differences between the current study and previous work [23]. Previously, there were significant decreases in basal VCAM-1 in micropatterned versus non-patterned ECs at 24 hours; however here we saw no discernible effect of micropatterning on TNF-α stimulated VCAM-1 expression. Additionally, in the previous work there had been an approximate doubling in KLF-2 expression in micropatterned ECs after 48 hours, while we saw only a very slight, and statistically non-significant increase in KLF-2 in micropatterned ECs after 50 hours here. There are three important factors which may explain these discrepancies: animal variability, TNF-α dosage and timing, and culture media serum content. Our previous study examined ECs isolated from a single primate, while this study used animal-matched cells from two different individuals, neither of which was included in the original study. Additionally, our previous study measured basal VCAM-1 expression [23], while we chose to stimulate VCAM-1 with a two-hour TNF-α treatment. This treatment is shorter than that used in our earlier flow cytometry characterizations of ECFC function [27], and was chosen based upon the results of pilot TNF-α time trials (data not shown). Additionally, serum content differed between these two studies. Previously, micropatterned cultures were fed with media containing only 2% serum, while we here used supplemented media totaling 10% serum. The earlier starvation conditions were motivated by a desire to slow cell division in order to keep ECs from expanding and growing over the BSA block dividing the patterned lanes. Because of the increased blocking efficacy of our polymeric blocking agent, and our desire to examine micropatterned cells at later time points, we deemed starvation unnecessary. However, because serum starvation arrests the cell cycle, it has an additional effect of amplifying observable changes to levels of gene transcription [37]. Thus, all changes in gene expression observed here may be lower in magnitude than what would be seen under the starvation conditions used previously. Given that we observed larger micropatterning effects in KLF-2 in ECFCs than ECs in this study, we would hypothesize that starvation conditions could replicate the prior findings, and demonstrate a greater level of micropattern-driven KLF-2 expression in ECFCs than ECs.

We found no significant micropatterning-driven changes in the expression of the pro-thrombotic von Willebrand factor (Fig 7). While we considered measuring vWF expression in TNF-α stimulated cells, the short cytokine insult period we chose to maximize VCAM-1 expression was not sufficient to produce a robust vWF response in our pilot studies (data not shown). It is possible that a vWF response could be seen coupled with a longer TNF-α treatment. Previous studies of the impact of fluid shear on vWF gene expression have been inconclusive. Early microarray experiments looking for flow-sensitive genes in ECs found that human umbilical vein ECs show a roughly 2-fold increase in vWF gene expression after seven days of fluid shear stress [15]. However a more recent study comparing human umbilical artery ECs, umbilical vein ECs, and ECFCs found no effect on vWF after 5 hours of fluid shear stress [38]. Interestingly, this study shows an increase in vWF expression in ECFCs compared to mature ECs after 24 hours of culture (Fig 7A vs. 7B, not evaluated statistically). While this finding might suggest an increase in basal thrombogenicity in ECFCs relative to ECs, it is important to note that our mature ECs are collected from carotid arteries, which have previously been shown to be areas of low vWF expression and production relative to other parts of the mammalian vascular tree [39,40]. Therefore, without a more anatomically diverse and representative pool of mature ECs to use as a comparison group, these results may not be indicative of an ECFC-specific enrichment in vWF expression compared to all mature ECs.

Static micropatterning can be broadly divided into techniques based on spatial growth restriction, and those based on topography. Spatial growth restriction, as shown here, is accomplished through the iterative adsorption of extracellular matrix molecules (which promote adhesion) and hydrophobic proteins or polymers (which block cell attachment) into lanes on the order of 10–30 μm (one cell width) wide using soft lithography techniques such as microcontact printing [21] or microfluidics (as here) [19]. In contrast, topographical micropatterning generates culture substrates containing ridges and grooves with subcellular pitches on the order of 0.4–10 μm [24]. The results of this spatial growth restriction study support the hypothesis that ECFC micropatterning could be a beneficial feature of tissue engineered artificial vascular grafts. We chose to use a spatial growth restriction method of micropatterning for this study because of its relative methodological ease, low material cost, and its relation to our earlier body of work [19,20,23,25]. This method is a valuable tool for basic science, however it has some important limitations which would hinder its translation to clinical applications. Spatial growth restriction requires the maintenance of non-endothelialized spacer regions between lanes which would expose the underlying biomaterial and defeat the purpose of an endothelialized vascular device. Second, this technique requires the establishment of a biochemically heterogeneous surface, which would be difficult to manufacture and maintain under prolonged contact with blood and tissue. Finally, the inter-lane divisions in the current approach prevent the full engagement of EC and ECFC cadherins which allow the formation of tight junctions and trigger the contact inhibition of cell division. Thus, to translate this work to tissue engineered artificial vascular grafts, future work should focus on whole graft patterning of ECFCs, similar to what other groups have accomplished with ECs [24,41–43]. This approach would drive the elongation of ECFCs in a confluent monolayer, which could result in greater durability under blood flow and a more physiologically relevant functional response.

This study also did not examine several features critical to the *in vivo* performance of ECFCs in vascular devices, including underlying pathology, substrate mechanical compliance and material thrombogenicity. We chose to study ECFCs from young, healthy primates, however there is evidence that EPCs and ECFCs function differently in patients with certain cardiovascular diseases [44]. Additionally, as described above, we chose to study spatial growth restriction on rigid polystyrene because of its relatively low cost and reproducibility. However, recent evidence suggests that matrix stiffness is associated with increased thrombogenic risk at the cellular level, necessitating attention to material mechanical properties when considering the design of novel vascular devices [45]. Substrate stiffness may have been a cause of the disruption seen to some of the VE-Cadherin junctions in Fig 2. Additionally, it is well known that collagen-I, which was used to promote EC and ECFC attachment in this study, is inherently thrombogenic and therefore not suitable for integration into blood-contacting devices [46]. Ongoing research into novel biomaterials with tunable mechanical properties capable of integrating less thrombogenic pro-endothelial peptide fragments such as GFPGER is addressing these issues [47]. This study provides evidence that anisotropic basal micropatterning could assist in the incorporation of ECFCs onto these and similar materials.

## Conclusions

Small-diameter artificial vascular grafts remain a critical unmet need. As interest in ECFCs as a potential tool for tissue engineered grafts grows, this study supports the use of this cell type for *in vitro* device endothelialization, particularly on micropatterned devices. These results demonstrate that ECFCs can be morphologically manipulated in the absence of fluid shear stress using spatial growth restriction micropatterning. Micropatterned ECFCs showed robust and lasting changes in gross and subcellular morphology analogous to that shown previously and repeated here with mature ECs. While the gene expression results shown here were largely unaffected by micropatterning, it should be noted that this study supports the use of ECFCs as equivalent to mature ECs in regulating neointima formation through the expression of adhesion molecules.

## Supporting information

S1 Dataset Raw data. Sheet 1 includes measured areas and perimeters, and calculated shape index values for traced cells (corresponding to the results shown in Fig 1). Sheet 2 includes values for each counted ROI for cell density and pyknosis studies, and an unblinding key (corresponding to Fig 3). Sheet 3 includes calculated ΔCT and -ΔΔCT values for gene expression experiments (corresponding to the results shown in Figs 4, 5, 6 and 7).
(XLSX)
